# Expression of microRNA-223 and microRNA-146b in serum and liver tissue of mice infected with *Schistosoma mansoni*

**DOI:** 10.1007/s00436-022-07542-3

**Published:** 2022-05-16

**Authors:** Hend A El-Taweel, Yasmine A Issa, Rasha F Mady, Ghada A Shehata, Eman A Youssef, Mona M Tolba

**Affiliations:** 1 Department of Parasitology, Medical Research Institute, Alexandria University, Alexandria, Egypt; 2 Department of Medical Biochemistry, Alexandria University, Alexandria, Egypt; 3 Department of Medical Parasitology, Faculty of Medicine, Alexandria University, Alexandria, Egypt; 4 Department of Histochemistry and Cell Biology, Medical Research Institute, Alexandria University, Alexandria, Egypt

**Keywords:** *Schistosoma mansoni*, Granulomas, Liver fibrosis, miRNA-223, miRNA-146b, Praziquantel

## Abstract

MicroRNAs (miRNAs) play regulatory roles in several diseases. In schistosomiasis, the main pathological changes are caused by the granulomatous reaction induced by egg deposition. We aimed to study the changes in host miRNA-223 and miRNA-146b expression in relation to egg deposition and development of hepatic pathology in murine schistosomiasis mansoni. Blood and liver tissue samples were collected from non-infected mice (group I), *S. mansoni*–infected mice at the 4th, 8th, and 12th weeks post-infection (p.i.) (groups II–IV), and 4 weeks after praziquantel treatment (group V). The collected samples were processed for RNA extraction, reverse transcription, and real-time PCR analysis of miRNA-223 and miRNA-146b. miRNAs’ relative expression was estimated by the ΔΔC_t_ method. Liver tissue samples were examined for egg count estimation and histopathological evaluation. Results revealed that miRNA-223 was significantly downregulated in liver tissues 8 and 12 weeks p.i., whereas miRNA-146b expression increased gradually with the progression of infection with a significantly higher level at week 12 p.i. compared to week 4 p.i. Serum expression levels nearly followed the same pattern as the tissue levels. The dysregulated expression of miRNAs correlated with liver egg counts and was more obvious with the demonstration of chronic granulomas, fibrous transformation, and distorted hepatic architecture 12 weeks p.i. Restoration of normal expression levels was observed 4 weeks after treatment. Collectively, these findings provide new insights for in-depth understanding of host-parasite interaction in schistosomiasis and pave a new way for monitoring the progress of hepatic pathology before and after treatment.

## Introduction

Schistosomiasis is a disease caused by trematodes belonging to the genus *Schistosoma* (Colley et al. 2014). It mainly burdens poor communities with inadequate access to safe water and sanitation in tropical areas (M'Bra et al. 2018; WHO 2020). During their life cycle, immature worms reach the hepatoportal circulation where they undergo sexual development within 6 weeks. Mature worms of *Schistosoma mansoni* and *Schistosoma. japonicum* live in the mesenteric veins and start to lay eggs, some of which are carried to the liver where they induce chronic granulomatous reaction (Davis 2009; Colley et al. 2014). The continuous production of eggs by adult female worms results in a massive increase in the number of eggs lodged in the liver and other tissues*.* (Von Lichtenberg et al. 1973). The hepatic stellate cells (HSCs) then differentiate into myofibroblasts initiating hepatic fibrosis, a major complication in schistosomiasis (Kamdem et al. 2018).

The discovery of microRNAs (miRNAs) about two decades ago represents a major advance in understanding the complex gene regulatory networks in biological processes (Lee et al. 1993). These short non-coding RNAs which are found in a wide range of body fluids and tissues control the expression of about 60% of protein-coding genes in man. Their action is mediated through directing target messenger RNA for translational repression or degradation (Chen et al. 2008; Huntzinger and Izaurralde 2011; O'Brien et al. 2018). The expression pattern of circulatory miRNAs is potentially considered a significant biomarker in the diagnosis and follow-up of various pathological states (Condrat et al. 2020). Blocking specific miRNA and/or improving the expression of others were tested as therapeutic strategies with promising results (Rupaimoole and Slack 2017).

In schistosomiasis, a variety of host miRNAs’ regulatory functions have been demonstrated (Cai et al. 2013; Hoy et al. 2014). The pattern of expression of hepatic miRNAs in patients with schistosomiasis differs from that of other hepatic diseases (Wang et al. 2012; Morishita et al. 2021). Throughout the course of *S. japonicum* infection, it was documented that the expression of some miRNAs in the liver are downregulated while the expression of other miRNAs is dramatically upregulated (Cai et al. 2013; Han et al. 2013; Cai et al. 2015; Kitano and Bloomston 2016; Cai et al. 2018; Tao et al. 2018; Zhao et al. 2019). Studies addressing miRNA expression in schistosomiasis mansoni are relatively limited.

Numerous miRNAs have been identified and were shown to modulate cell proliferation, differentiation, metabolism, tissue remodeling, immunity, and intracellular signaling (de Rie et al. 2017). miRNA-223 is a crucial factor in the activation and homeostasis of immune functions (Haneklaus et al. 2013; Yuan et al. 2018). It is produced by Kupffer cells in the liver (He et al. 2013). Increasing evidence has revealed that miRNA-223 is implicated in the pathogenesis of several liver diseases through regulation of immune cell differentiation, macrophage polarization, neutrophil infiltration, inflammasome activation, and metabolic signaling pathways (Ye et al. 2018). miRNA-146b is another key regulator of the host immune response. It has a role in the control of Toll-like receptors and cytokine signaling (Taganov et al. 2006). Activation of miRNA-146b following antigen recognition facilitates a negative-feedback loop that protects the host from an excessive inflammatory response (Taganov et al. 2006; Schulte et al. 2013). miRNA-146b is also involved in regulating HSC activation that modulates hepatic fibrosis (Ge et al. 2016).

The present work aimed to investigate the dynamics of miRNA-223 and miRNA-146b expression in relation to egg deposition and development of hepatic pathology along the course of *S. mansoni* infection in mice.

## Material and methods

### Experimental design

Pathogen-free female Swiss albino mice, 4 to 6 weeks old, weighing 20–22 g were used in the study. They were bred in stainless steel wire-mesh cages under controlled conditions (temperature 18–25°C, humidity 30–60%, 12-h light/dark cycles). The use of mice followed the Ethical guidelines of the Institutional Animal Care and Use Committee (IACUC), Alexandria University, Egypt (number: AU01218123021). The study complies with ARRIVE guidelines and the National Research Council guide for the care of laboratory animals.

The mice were divided into five groups, each comprised six mice:

- Group I: non-infected mice
- Group II: infected, sacrificed 4 weeks post-infection (p.i.)
- Group III: infected, sacrificed 8 weeks p.i.
- Group IV: infected, sacrificed 12 weeks p.i.
- Group V: infected, treated 8 weeks p.i. by a praziquantel (PZQ) oral dose of 500 mg/kg/day for 2 days (El-Lakkany et al. 2012) and sacrificed 12 weeks p.i. (4 weeks post-treatment).

At the time of sacrifice, peripheral blood samples were collected, and sera were separated and tested for miRNA expression. Liver samples were obtained from the left lateral lobe for assessment of miRNA expression, tissue egg count estimation, and histopathological examination.

### Induction of S. mansoni infection

Laboratory-bred *Biomphalaria alexandrina* snails were infected with the miracidia of *S. mansoni* Egyptian strain at the *Schistosoma* Biological Supply Program (SBSP), Theodor Bilharz Research Institute, Giza, Egypt. The snails were washed with dechlorinated tap water (DTW) to remove excreta and other debris. Cercarial shedding was induced by placing snails in DTW and exposing them to direct sunlight for 30–60 min (Liang et al. 1987). Mice were infected by the tail immersion technique where each mouse was separately exposed to 50 *S. mansoni* cercariae in 10 ml DTW for 1 h at room temperature (Smithers and Terry 1965). Each infected group comprised six mice. The establishment of infection was confirmed in groups III–V by detection of *S. mansoni* eggs in stool samples collected 7 weeks p.i.

### Egg count estimation and histopathologic examination

*S. mansoni* egg count per gram of liver tissue was estimated in each mouse using a digestion technique. Briefly, 1 g of liver tissue was kept overnight in 5 mL potassium hydroxide solution (4%). After thorough mixing, *S. mansoni* eggs in three drops of the suspension (each measure 0.1ml) were counted. The hepatic egg load was expressed as egg per gram (epg) (Cheever 1968).

Specimens of the liver tissue were fixed in 10% neutral formalin for hematoxylin and eosin (H&E) staining and histopathological examination (Kiernan 1999). Histomorphometric examination was performed using Image J software (Java-based application for analyzing images) to measure the average granuloma diameter (μm) in infected mice. A total of 25 granulomas were examined in each group (Yepes et al. 2014). Digital images were randomly obtained in 10–20 fields per group at a magnification of 100x (Fischer et al. 2008).

### miRNA-223 and miRNA-146b expression

Total RNA was isolated from serum and liver samples of mice using miRNeasy Mini Kit (QIAGEN, Maryland, USA, Cat No. 217004) according to the manufacturer’s instructions. The concentration and purity of RNA were measured at 260, 280, and 230 nm using NanoDrop 2000 Spectrophotometer (Thermo Scientific, USA). A 260:230 ratio greater than 1.7 and a 260:280 ratio greater than 2.0 indicates highly pure RNA.

RNA was reverse-transcribed using the high-capacity cDNA synthesis kit (TaqMan® MicroRNA Reverse transcription Kit Catalog no.4366596) and miRNA primers supplied with the TaqMan® miRNA assay (Bustin and Mueller 2005). The thermal cycler was set at 16°C for 30 min, 42°C for 30 min, and 85°C for 5 min, then the temperature was lowered to 4°C and the run was stopped. Complementary DNA (cDNA) was stored at −20°C until performing real-time qPCR.

Real-time qPCR was performed on Applied Biosystems TaqMan® Universal Master Mix II, using specific primers supplied by TaqMan® MicroRNA Assays (mmu miRNA-223, mmu miRNA-146b) catalog no. 4428175, assay I.D 002253, and catalog no. 4427975, assay ID 002453 respectively. Negative control samples were added to rule out contamination. A housekeeping gene (HKU6) was used as the endogenous reference gene for normalization. All samples were run in duplicates. Real-time qPCR steps included an initial cycle of 95°C for 10min followed by 40 cycles of 95°C for 15s, and finally, the temperature was lowered to 60°C for 1min. Fluorescent signals from each sample were collected at the endpoint of every cycle (Arya et al. 2005). The resulting cycle threshold (*C*_*t*_) values of the tested samples were determined. The fold change in miRNA-223 and miRNA146b expression was calculated using the relative quantification (RQ) method where Δ*C*_*t*_ sample = *C*_*t*_^miRNAtarget^ −*C*_*t*_^HK^, ΔΔ*C*_*t*_ = Δ*C*_*t*_ sample − average Δ*C*_*t*_ non-infected group and RQ = 2 ^−ΔΔ*Ct*^. Fold change values greater than 1 indicate upregulated expression, while fold-change values less than 1 indicate downregulated expression (Livak and Schmittgen 2001).

### Statistical analysis

Data analysis was performed using IBM SPSS software package version 20.0 (Armonk, NY: IBM Corp). The Kolmogorov-Smirnov test was used to verify the normality of distribution. Normally distributed quantitative data were presented as mean ± standard deviation (SD) and compared using the *F*-test (one-way analysis of variance, ANOVA) followed, in case of significance, by the post hoc test (Tukey). Data that do not follow normal distribution were presented as minimum-maximum and median and compared using the Kruskal-Wallis test followed, in case of significance, by the post hoc (Dunn’s multiple comparisons test). The Spearman coefficient was calculated to study the correlation between quantitative variables. The level of statistical significance was set at 5%.

## Results

### Hepatic egg count and granuloma size

Mice euthanized 4 weeks p.i. (group II) had neither eggs nor hepatic granulomas in liver tissues. There was a statistically significant difference between the other infected groups regarding hepatic egg counts and granuloma size as demonstrated by the *F* test (ANOVA) (egg count: *p*<0.001, granuloma size: *p*= 0.012). Pairwise comparison showed that group IV (12 weeks p.i) had significantly higher mean egg count/g of liver tissue compared to group III (8 weeks p.i.) (*p* <0.001). However, the two groups showed a non-significant difference in granuloma size. The treated mice (group V) had significantly lower mean egg count and significantly smaller granuloma size compared to group III (*p*< 0.001 and *p*= 0.048 respectively) and group IV (*p*<0.001 and *p*=0.016 respectively) (Table 1).

**Table 1:** Hepatic egg count and granuloma size in the infected mice groups

	**Group III**	**Group IV**	**Group V**	***F***	***p***
**Egg count/g liver tissue (×10**^**2**^**)**					
Min.–max.	4.68–6.26	11.80–16.20	1.11–1.74	351.49	<0.001*
Mean ± SD	5.59 ± 0.67	14.11 ± 1.50	1.45 ± 0.24		
Pairwise comparison	***p***_**1**_<0.001*, ***p***_**2**_<0.001*, ***p***_**3**_<0.001*				
**Granuloma size**^a^ **(in μm)**					
Min.–max.	131.94–383.72	150.99–523.32	105.41–318.56	4.71	0.012*
Mean ± SD	271.66 ± 63.88	280.76 ± 94.71	221.30 ± 57.35		
Pairwise comparison	p_1_=0.901, **p**_**2**_=0.048^*^, p_**3**_=0.016^*^				

*F*: *F* for ANOVA test, pairwise comparison between each two groups was done using post hoc test

***P***_**1**_: *p* value for comparing between group III and group IV, ***P***_**2**_: *p* value for comparing between group III and group V, ***P***_**3**_: *p* value for comparing between group IV and group V

*Statistically significant at *p* ≤ 0.05

**Group III**: scarified 8 weeks p.i, **Group IV**: scarified 12 weeks p.i, **Group V**: treated by PZQ 8 weeks p.i. and scarified 12 weeks p.i. No eggs were detected in group II (4 weeks p.i.)

^a^25 granulomas were examined in each group

### Histopathological examination

**Fig. 1:**
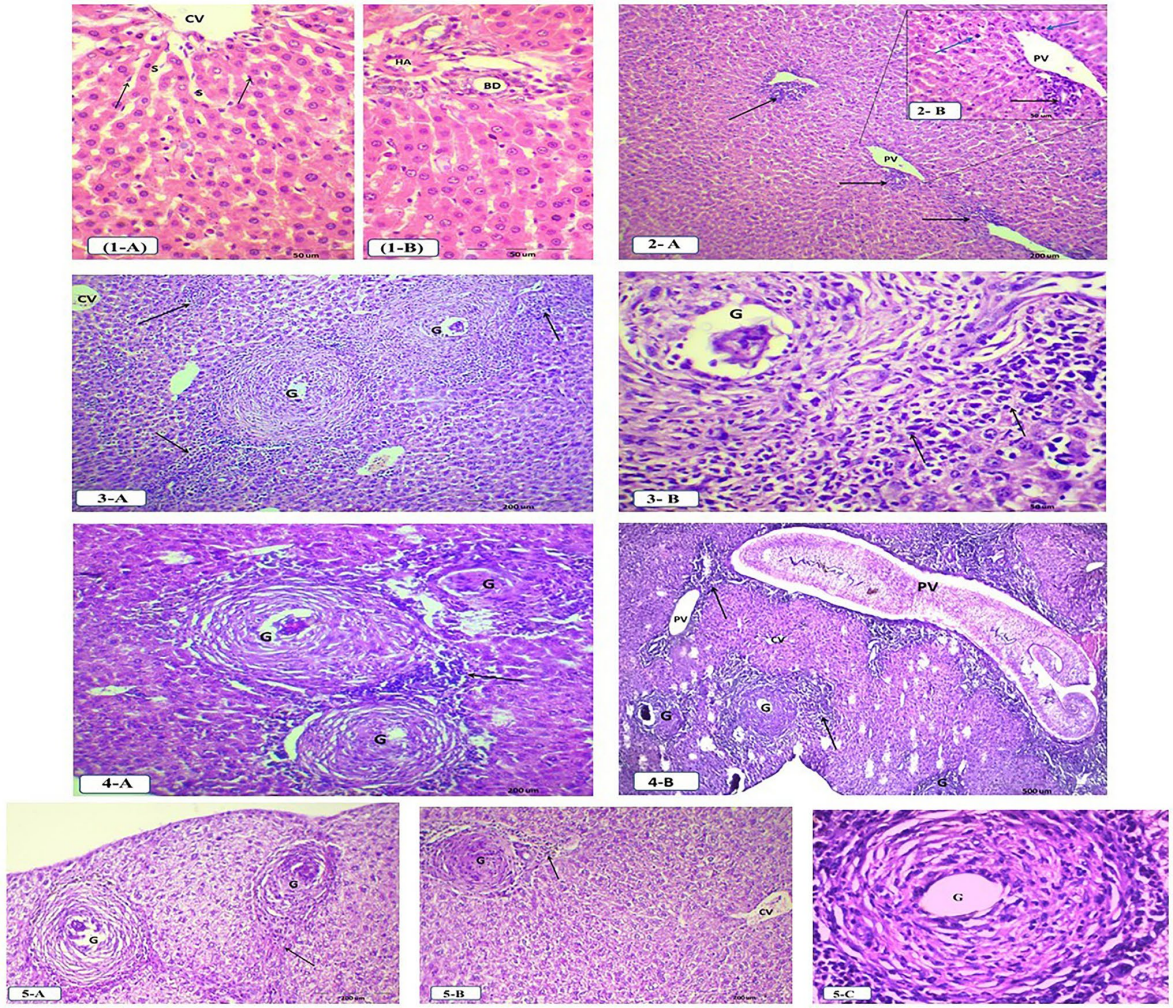
Photomicrographs of H&E-stained liver sections of the studied mice groups. (**1-A** and **1-B**) Liver sections of group I mice (normal control) showing hepatic sinusoids (S) between the adjacent plates attached with central vein (CV) and Kupffer cells (↑) associated with the sinusoidal lining cells (**1-A**) and Portal tract containing branches of hepatic artery (HA) and bile duct (BD) (**1-B**) (H&E-Bar=50 μm). (**2-A** and **2-B**) Liver sections of group II mice (4 weeks p.i) showing obvious inflammatory cellular infiltration especially in periportal area (↑) (**2-A**) (H&E-Bar=200 μm) and inflammatory cellular infiltration (↑) around portal vein (PV) in addition to apoptosis in some hepatocytes (blue arrow) (**2-B**) (H&E-Bar=50 μm). (**3-A** and** 3-B**) Liver sections of group III mice (8 weeks p.i) showing large hepatic granuloma (G), increase in inflammatory cellular infiltration in portal area (↑) and branch of central vein (CV) (**3-A**) (H&E-Bar=200 μm) and granuloma (G) with epithelioid cells around mature egg, middle zone of fibroblasts and fibrocytes and cellular infiltrations in outermost zone (↑) (**3-B**) (H&E-Bar=50 μm). (**4-A** and **4-B**) Liver sections of group IV mice (12 weeks p.i) showing distorted hepatic architecture with chronic fibrous granulomas (G) around a mature egg and inflammatory cellular infiltration (↑) (**4-A**) (H&E-Bar=200 μm) and dilated portal vein (PV) with trapped adult worm, chronic inflammatory cellular infiltration in periportal area (↑), many granulomas (G) and a branch of the central vein (CV) (**4-B**) (H&E-Bar=500 μm). (**5-A**, **5-B**, and **5-C**) Liver sections of group V mice (4 weeks post PZQ treatment) showing fibrotic appearance of a granuloma (G) with more reduced size and fibrous septa (↑) (**5-A**) (H&E-Bar=200 μm), noticeable improvement in hepatic lobule with hepatocytes radiating from the central vein (CV) along with fibrous granuloma (G) and diminished inflammatory cellular infiltration (↑) (**5-B**) (H&E-Bar=200 μm) and fibrotic granuloma with no eggs (**5-C**)

Liver sections of non-infected mice revealed obvious hepatic sinusoids with hepatocytes arranged in plates radiating from the central vein. Kupffer cells were seen associated with the sinusoidal lining cells, and portal tract containing branches of the hepatic artery and bile duct were seen at the periphery of the liver lobules (Fig. 1(1A, 1B)).

Histopathologic examination of the liver tissue four weeks p.i. (group II) revealed evidence of inflammatory cellular infiltration, especially in the portal tracts with apoptosis in some hepatocytes (Fig. 1(2A, 2B)). Mice of group III (8 weeks p.i.) showed large hepatic granulomas around mature eggs with epithelioid cells in the inner zone, fibroblasts, and fibrocytes in the middle zone, and cellular infiltrations consisting of lymphocytes, plasma cells, and eosinophils in the outermost zone. An increase in inflammatory cellular infiltration was observed in the portal area (Fig. 1(3A, 3B)).

Mice were sacrificed 12 weeks after infection (group IV) displayed distorted hepatic architecture with many chronic granulomatous lesions in the hepatic parenchyma and fibrous transformation of granulomas around mature eggs (Fig. 1(4A)). Dilated portal vein with a trapped adult worm and chronic inflammatory cellular infiltration was observed at low power magnification (Fig. 1(4B)).

Liver sections of PZQ-treated mice showed a more fibrotic appearance of granulomas with a reduction in their size (Fig. 1(5A)). Compared to the infected non-treated group, mice treated with PZQ had improvement in hepatic lobule with diminished inflammatory cellular infiltration in the periportal area, and fibrous granulomas with either no eggs or degenerated eggs (Fig. 1(5B, 5C)).

### miRNA-223 expression

Kruskal-Wallis test revealed a statistically significant difference between the studied groups regarding miRNA-223 expression in the liver tissue (*H*= 23.407, *p* <0.001) and serum samples (*H*=13.828, *p*= 0.008). Significant downregulation of miRNA-223 was observed in the liver tissues and serum samples of the infected untreated mice at week eight (*p*= 0.019 and *p* =0.032 respectively) and week 12 p.i. (*p*=0.001) compared to the uninfected mice. Expression levels at week 12 were significantly lower than the expression at week 4 in both liver tissue (*p*=0.009) and serum samples (*p*=0.004) (Table 2).

**Table 2:** miRNA-223 relative expression level in the liver tissue and serum samples of the studied groups

**RQ**	**Group I** Non-infected	**Group II** (4 weeks p.i.)	**Group III** (8 weeks p.i.)	**Group IV** (12 weeks p.i.)	**Group V** (PZQ treated)	***H***	***p***
**Tissue**							
Min.–max.	1.0–1.2	0.21–0.91	0.08–0.48	0.02–0.07	0.90–1.06	23.407	<0.001*
Median	1.05	0.87	0.20	0.03	0.96		
Pairwise comparison							
*p*_1_		0.181	0.019*	0.001*	0.953		
*p*_2_			0.216	0.009*	0.118		
*p*_3_				0.167	**0.005**		
*p*_4_					<0.001*		
**Serum**							
Min.–max.	1.0–1.1	0.71–0.99	0.31–0.70	0.11–0.42	0.12–2.02	13.828	0008*
Median	1.0	0.89	0.47	0.25	0.79		
Pairwise comparison							
*p*_1_		0.365	0.032*	0.001*	0.082		
*p*_2_			0.131	0.004*	0.308		
*p*_3_				0.178	0.623		
*p*_4_					0.066		

RQ: Relative quantification, H: H for Kruskal-Wallis test, *p*: *p* value for comparing between the different studied groups. Pairwise comparison between each two groups was done using post hoc test (Dunn’s for multiple comparisons test)

*p*_1_: *p* value in comparison to group I

*p*_2_: *p* value in comparison to group II

*p*_3_: *p* value in comparison to group III

p_4_: *p* value in comparison to group IV

*Statistically significant at *p* ≤ 0.05

The expression levels in the treated mice (group V) were not significantly different from the corresponding pre-infection (group I) and early infection (group II) levels in tissue and serum samples. The expression in the liver tissue (but not serum samples) of the treated mice was relatively high compared to the levels observed at week 8 p.i. (*p* = 0.005) and week 12 p.i (*p*<0.001) (Table 2).

**Fig. 2:**
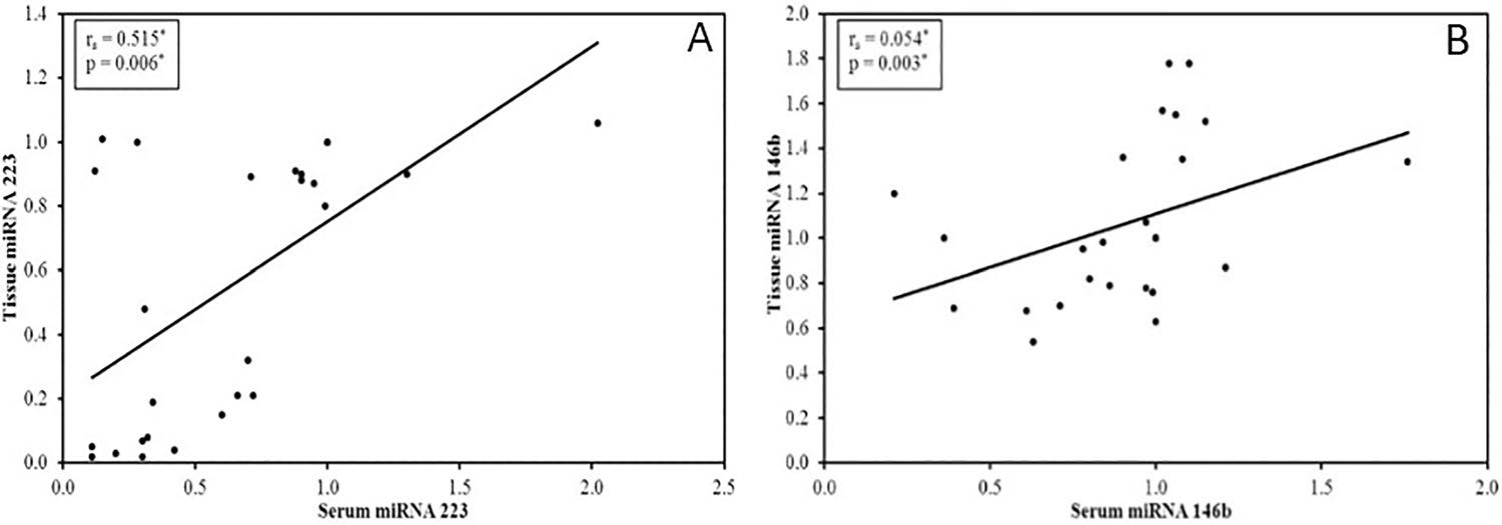
Correlation between serum and tissue levels of miRNA-223 (**A**) and miRNA-146b (**B**)

**Fig. 3:**
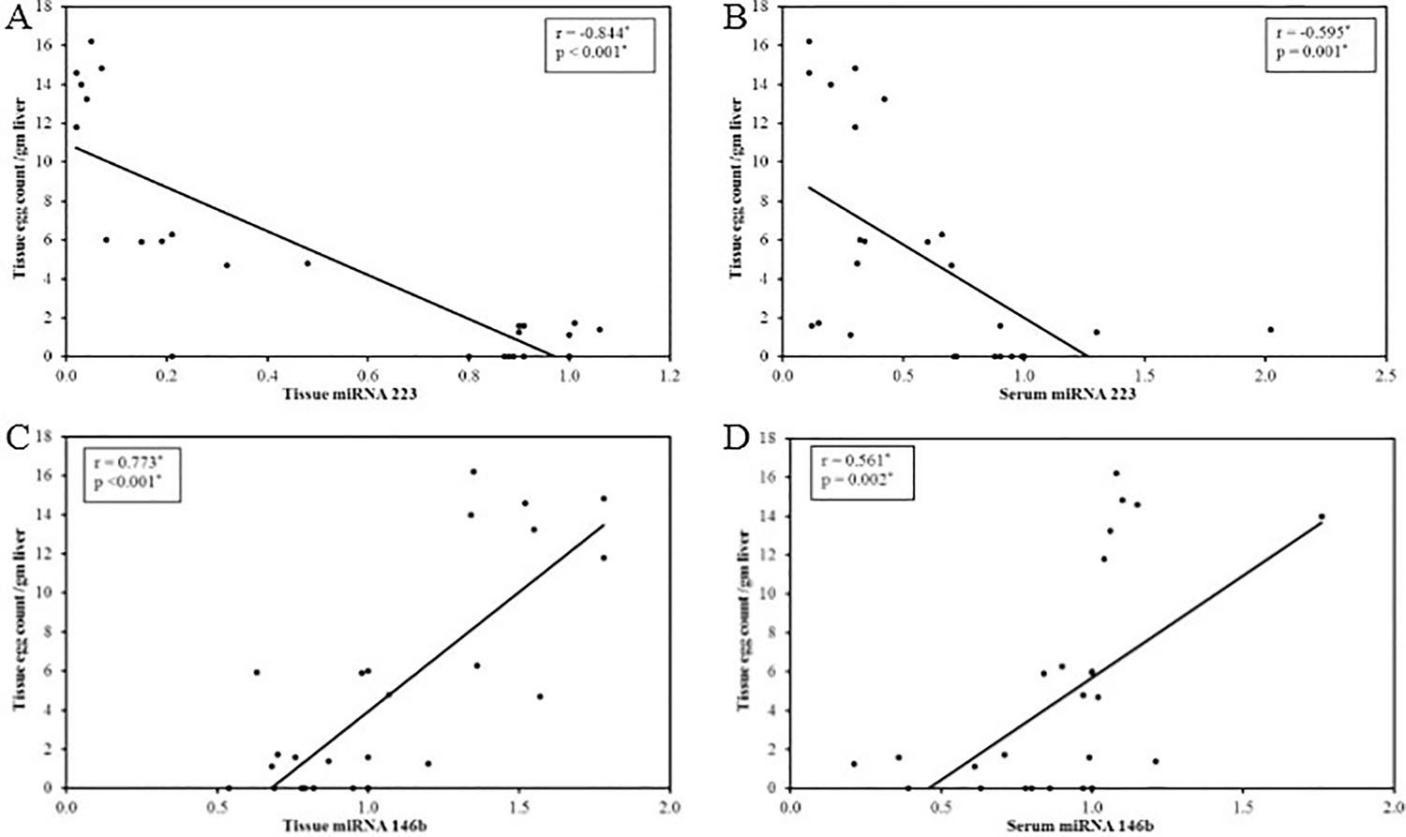
**A**–**D** Correlation between miRNA levels and hepatic egg counts

A significant positive correlation was detected between tissue miRNA-223 and serum miRNA-223 expression (*r*_s_ = 0.515, *p*= 0.006) (Fig. 2A) and both correlated negatively with hepatic egg count (*r*_s_ = −0.844, *p* =< 0.001 and *r*_s_ = −0.595, *p* = 0.001 respectively) (Fig. 3A, B).

### miRNA 146b expression

Kruskal-Wallis test revealed a statistically significant difference between the studied groups regarding miRNA-146b expression in the liver tissue (*H*= 15.767, *p* = 0.003) and serum samples (*H*= 15.720, *p* = 0.003). There was no significant difference between the non-infected and infected mice euthanized at any stage of infection. At week 12, however, significant upregulation was observed in both tissue and serum samples compared to week 4 (*p*<0.001) and in serum samples compared to week 8 (*p* = 0.042). Treated mice showed significantly lower miRNA-146b expression levels in liver tissues and serum samples in comparison to mice euthanized 12 weeks p.i. (*p* = 0.003 and 0.001 respectively) (Table 3).

**Table 3:** miRNA-146b relative expression level in the liver tissue and serum samples of the studied groups

**RQ**	**Group I** (Non-infected)	**Group II** (4 weeks p.i.)	**Group III** (8 weeks p.i.)	**Group IV** (12 weeks p.i.)	**Group V** (PZQ treated)	***H***	***p***
**Tissue**							
Min.–max.	0.8–1.2	0.54–0.95	0.63–1.57	1.34–1.78	0.68–1.20	15.767	0.003*
Median	1.0	0.78	1.04	1.53	0.82		
Pairwise comparison							
*p*_1_		0.115	0.976	0.153	0.305		
*p*_2_			0.058	<0.001*	0.500		
*p*_3_				0.074	0.222		
*p*_4_					0.003*		
**Serum**							
Min.–max.	0.9–1.1	0.39–0.97	0.84–1.02	1.04–1.76	0.21–1.21	13.828	0.008*
Median	1.0	0.79	0.98	1.09	0.66		
Pairwise comparison							
*p*_1_		0.096	0.634	0.235	0.145		
*p*_2_			0.145	<0.001*	0.799		
*p*_3_				0.042*	0.230		
*p*_4_					0.001*		

RQ: Relative quantification. H: H for Kruskal-Wallis test, *p*: *p* value for comparing between the different studied groups. Pairwise comparison between each two groups was done using post hoc test (Dunn’s for multiple comparisons test)

*p*_1_: *p* value in comparison to group I

*p*_2_: *p* value in comparison to group II

*p*_3_: *p* value in comparison to group III

*p*_4_: *p* value in comparison to group IV

*Statistically significant at *p* ≤ 0.05

A significant positive correlation was detected between tissue miRNA-146b and serum miRNA-146b expression (*r*_s_ = 0.054, *p*< 0.003) (Fig. 2B) and both correlated positively with hepatic egg count (*r*_s_ = 0.773, *p*< 0.001and *r*_s_ = 0.561, *p*= 0.002 respectively) (Fig. 3C, D).

## Discussion

Altered microRNA expression has been linked to the development of several diseases (Li and Kowdley 2012; Bartoszewski and Sikorski 2018). The present study revealed stage-dependent downregulation of miRNA-223 expression during *S. mansoni* infection in mice. Suppression of miRNA-223 occurred at week 8 p.i. coinciding with egg deposition and formation of hepatic granulomas. Expression correlated negatively with tissue egg count and was more obvious with the progression of infection and appearance of chronic granulomatous lesions, fibrous transformation, and distorted hepatic architecture in liver sections. Serum miRNA-223 expression followed the same pattern as the tissue level in untreated mice and was inversely related to the progression of histopathological damage. Accordingly, circulating miRNA-223 can be considered a non-invasive indicator of schistosomal hepatic pathology after excluding diseases with similar pathogenic processes of inflammation and fibrosis (Ye et al. 2018).

Contrary to our findings, He et al. (2013) found that the expression of miRNA-223 was significantly upregulated in the hepatocytes, HSCs, and Kupffer cells in *S. japonicum*–infected mice. Moreover, they reported that the level of circulating miRNA-223 increased significantly and correlated positively with the egg burden and hydroxyproline content in the liver tissue. The discrepancy between *S. japonicum* and *S. mansoni* in miRNA-223 expression may be explained by differences in the immunopathology of the two species. *S. mansoni* egg-induced granulomas are primarily formed of eosinophils with a low neutrophils content, whereas *S. japonicum* egg-induced granulomas consist mainly of neutrophils (Von Lichtenberg et al. 1973). Furthermore, *S. mansoni* eggs can inhibit the differentiation of HSCs into myofibroblasts and can reverse the trans-differentiation or activation process to return HSCs to their quiescent state (Anthony et al. 2010). By contrast, *S. japonicum* eggs activate the pro-inflammatory phenotype of HSCs (Anthony et al. 2013).

Regarding the liver expression of miRNA-146b, we found that it increased gradually as the infection progresses resulting in a significantly higher level in mice examined in the latter stage of infection (12 weeks p.i.) compared to mice examined earlier (4 weeks p.i.) although the difference between infected and control mice was statistically non-significant. Unlike miRNA-223, expression of miRNA-146b correlated positively with egg count. In agreement with these results, Cai et al. (2013) observed that the expression pattern of miRNA-146b in liver tissue did not change dramatically during the early prepatent stage of schistosomiasis japonicum in mice. However, upregulation of miRNA-146b occurred 30 days p.i. and increased thereafter, when hepatic granulomatous reaction and fibrosis became more profound. Upregulation of miRNA-146b in response to *S. japonicum* infection was demonstrated by He et al. (2015) using miRNAs’ microarrays. In another study, He et al. (2016) found that miRNA-146b is mainly expressed by hepatic macrophages 6 weeks p.i. in *S. japonicum*–infected mice. Upregulation of miRNA-146b synchronizes with the transition from a Th1 to a Th2 response following egg deposition. The release of Th2 cytokines such as interleukin-10 (IL-10), IL-4, and IL-13 enhances the activity of signal transducer and activator of transcription-3 which binds to the promoter of the pre-miRNA-146b gene and upregulates miRNA-146b expression. Meanwhile, IL4 and Il-13 enhance macrophage differentiation to M2 cells which promote the synthesis of liver collagen and subsequent hepatic fibrosis (Ding et al. 2019).

The association between miRNA dysregulated expression and the onset of egg deposition and granulomatous reactions was previously verified in other miRNAs. Hoy et al. (2014) reported an increase in miRNA-199-5p, miRNA-199-3p, miRNA-214, miRNA-21, and miRNA-210 and a reduction of miRNA-192 miRNA-122 and miRNA-194 expression in infected mice by 6–8 weeks p.i. The changes were augmented thereafter denoting that miRNAs have key roles in the development and progression of liver fibrosis. In another study, Cai et al. (2015) observed differential expression of miRNA-122, miRNA-21, and miRNA-34a in mice strains exhibiting different degrees of schistosomal hepatic fibrosis.

We observed that miRNA-146b expression in serum samples showed a positive correlation with the liver expression levels and was significantly elevated in the advanced stage (12 weeks p.i.) compared to the two earlier follow-up periods (4 and 8 weeks p.i.). In contrast, previous studies in *S. japonicum* mice models demonstrated downregulation of circulatory miRNA-146b but upregulated hepatic expression (Cai et al. 2013; He et al. 2013). A possible explanation for this contradiction is that circulatory miRNAs are affected by expression in several tissues such as the intestine, spleen, kidney, and other ectopic sites, especially in schistosomiasis japonicum (Lima et al. 2017; Cai et al. 2018).

In addition to their potential use as a biomarker for diagnosis and determination of disease severity, miRNAs could be used as indicators for therapeutic effects (Correia et al. 2017; Wu et al. 2021). In the current study, the dynamics of miRNA expression were further scrutinized by examination of treated mice. It was found that PZQ treatment resulted in the restoration of tissue and serum miRNA-223 towards the normal levels. Importantly, tissue miRNA-223 expression was significantly higher in PZQ-treated mice compared to the untreated groups examined 8 or 12 weeks p.i. (groups III and IV). PZQ-treated mice also showed a significant reduction in liver and serum miRNA-146b expression compared to the group with untreated advanced infection. The changes in miRNA expression in treated mice were accompanied by lower hepatic egg counts, smaller hepatic granuloma size, and diminished inflammatory cellular infiltration. This is attributed to degeneration of trapped eggs and cessation of egg-laying and antigen release. In agreement with our findings, He et al. (2013) reported that the disturbed miRNA-223 expression in *S. japonicum*–infected mice turned into a level similar to the uninfected mice after elimination of infection by PZQ. Cai et al. (2018) reported that the considerable regression of liver fibrosis after PZQ treatment in *S. japonicum*–infected mice was associated with restoration of normal levels of six miRNAs (miR-146a-5p, miRNA-150-5p, miR-200b-3p, and miR-222, let-7a-5p, and let-7d-5p). The findings of the present study indicate that tissue miRNA-223 as well as serum and tissue miRNA-146b expression are sensitive to chemotherapy. Therefore, they can be used to monitor the therapeutic effects of PZQ in terms of improvement of hepatic histopathological damage in *S. mansoni*–infected host. Since the evaluation of tissue expression levels requires examination of a liver biopsy specimen, serum miRNA-146b would be preferred in clinical settings as a less invasive marker.

The present study highlights the pertinence between hepatopathological changes and expression of miRNA-223 and miRNA-146b during *S. mansoni* infection. The underlying mechanisms depend on miRNA targets. The miRNA-223 is a hematopoietic cell-specific miRNA that is highly expressed in circulating monocytes and neutrophils as well as tissue-infiltrated macrophages. In liver tissue, it is mainly produced by Kupffer cells (He et al. 2013). It plays crucial roles in preventing liver injury and fibrogenesis through regulating nuclear factor-κB dependent macrophage differentiation and inflammasome activation. miRNA-223 expression acts as a negative controller of severe inflammation where a suppressed level is associated with chronic inflammation (Haneklaus et al. 2013; Ye et al. 2018).

miRNA-146b expression is upregulated in macrophages by lipopolysaccharide stimulation through an IL-10-dependent pathway. Tumor necrosis factor α and interleukin-1β enhance its production. miRNA-146b activation contributes to regulation of the inflammatory responses (Taganov et al. 2006; Curtale et al. 2013; Schulte et al. 2013). miRNA-146b is also involved in regulating hepatic macrophage function. It blocks macrophage differentiation to M1 cells via the induction of signal transducer and activator of transcription-1. Since M1 macrophages form pro-inflammatory cytokines which enhance inflammation and tissue damage, it is likely that miRNA-146b plays an important role in the conversion of the acute inflammatory phase to a chronic one in schistosomiasis. miRNA-146b also modulates hepatic fibrosis by regulating the HSC activation pathway (He et al. 2016; Ge et al. 2016). Moreover, overexpression of miRNA-146b in fibroblasts was found to contribute to increased collagen content via targeting the tissue inhibitor of metalloproteinase (Ye et al. 2021).

Recently, the potential of using miRNA inhibitors or mimics to block or overexpress miRNA actions has been proposed as a therapeutic intervention for several diseases. In schistosomiasis, reduced severity of hepatic fibrosis was reported following inhibition of miRNA-21 (He et al. 2015). Results of the present study highlight the potential importance of miRNA-223 and miRNA-146b as candidates of anti-fibrotic therapy in schistosomiasis. In this regard, miRNA-146b knockdown was previously shown to decrease the proliferation of HSCs and reduce the synthesis of fibrogenic proteins (Ge et al. 2016). The therapeutic potential of the studied miRNAs still deserves further investigation.

In conclusion, the expression levels of miRNA-123 and miRNA-146b correlate with liver egg count and become more obvious in the advanced stages of infection. They can be used as markers to reflect and follow the extent of liver pathology. Collectively, the study findings provide new insights for the in-depth understanding of host-parasite interactions in schistosomiasis mansoni and pave a new way for monitoring the progress of hepatic pathology before and after treatment.
